# Get Out and Stay Out: New Insights Into DNA Methylation Reprogramming in Mammals

**DOI:** 10.3389/fcell.2020.629068

**Published:** 2021-01-07

**Authors:** Maxim V. C. Greenberg

**Affiliations:** Centre National de la Recherche Scientifique, Institut Jacques Monod, Université de Paris, Paris, France

**Keywords:** DNA methylation, epigenetics, reprogramming, mammalian development, embryonic stem cells (ESC)

## Abstract

Vertebrate genomes are marked by notably high levels of 5-cytosine DNA methylation (5meC). The clearest function of DNA methylation among members of the subphylum is repression of potentially deleterious transposable elements (TEs). However, enrichment in the bodies of protein coding genes and pericentromeric heterochromatin indicate an important role for 5meC in those genomic compartments as well. Moreover, DNA methylation plays an important role in silencing of germline-specific genes. Impaired function of major components of DNA methylation machinery results in lethality in fish, amphibians and mammals. Despite such apparent importance, mammals exhibit a dramatic loss and regain of DNA methylation in early embryogenesis prior to implantation, and then again in the cells specified for the germline. In this minireview we will highlight recent studies that shine light on two major aspects of embryonic DNA methylation reprogramming: (1) The mechanism of DNA methylation loss after fertilization and (2) the protection of discrete loci from ectopic DNA methylation deposition during reestablishment. Finally, we will conclude with some extrapolations for the evolutionary underpinnings of such extraordinary events that seemingly put the genome under unnecessary risk during a particularly vulnerable window of development.

## Introduction

5-cytosine DNA methylation (5meC) is a modification conserved across all kingdoms of eukaryotes. Most generally it is found in the CpG dinucleotide context, and there are factors that ensure faithful daughter strand methylation after each round of DNA synthesis (Bostick et al., 2007; Sharif et al., 2007; Woo et al., 2008). Given the tight link with DNA replication, 5meC has high potential to exhibit epigenetic stability. In flowering plants, so-called “epialleles”—alleles that differ in 5meC content, not their DNA sequence—can persist for an indefinite number of generations (Weigel and Colot, 2012). Recently it was demonstrated in the yeast *Cryptococcus neoformans* that DNA methylation patterns have endured for millions of years exclusively through a maintenance mechanism, as there is an absence of *de novo* DNA methylation enzymes in the genome (Catania et al., 2020).

Standing apart from other eukaryotic lineages, it has been known for decades that mammals exhibit two rounds of dramatic DNA methylation reprogramming during embryonic development: first immediately after fertilizations, and a second time in the germline (Monk et al., 1987). The most facile explanation for the initial 5meC erasure event is that the embryo must level the high DNA methylation asymmetry exhibited by the paternal and maternal gametic genomes that arrive in the zygote (Wang et al., 2014), thus mitigating dosage discrepancies between alleles. By the blastocyst stage, which coincides with naive pluripotency, residual DNA methylation (~20% of CpGs) is largely restricted to genomic imprints and TEs (Wang et al., 2014, 2018; Zhu et al., 2018). As the embryo implants in the uterus, the *de novo* DNA methyltransferases, DNMT3A and DNMT3B, rapidly remethylate the genome to 70–80% CpG methylation, establishing a pattern that is globally maintained in somatic tissue-types (Borgel et al., 2010; Seisenberger et al., 2012; Smith et al., 2017; Zhang et al., 2018). This whole process repeats itself in primordial germ cells (PGCs), with a key difference being that in the germline, genomic imprints are erased and then reset in a sex-specific manner (Hajkova et al., 2002; Lee et al., 2002).

The last decade has seen advances in sequencing protocols and technology that have allowed for stunning temporal resolution of allele-specific DNA methylation patterns in early mammalian development (Wang et al., 2014, 2018; Gkountela et al., 2015; Zhu et al., 2018; Grosswendt et al., 2020). Nevertheless, much of the mechanistic underpinning of these processes remains lacking. To this end, embryonic stem cells (ESCs), which are derived from the inner cell mass (ICM) of the blastocyst, remain a powerful model for exploring the bases of the phenomenology of DNA methylation reprogramming. In this mini-review, we will highlight recent findings made in mouse ESCs (mESCs) that may help explain (1) how rapid global demethylation occurs, and (2) how promoters remain protected from the onslaught of DNA methylation establishment during implantation. Finally, given that DNA methylation reprogramming is a peculiarity—one that does not even appear to occur in our non-mammalian vertebrate cousins—we will discuss a possible clue that might explain the evolutionary rise of counterintuitive events.

## Passive Aggressive: DNA Demethylation After Fertilization

DNA demethylation can occur via either passive or active means. Passive demethylation simply requires the impairment of maintenance DNA methylation machinery, which results in 2-fold dilution of methyl-CpGs during each round of DNA synthesis. In mammals, active DNA demethylation occurs through action of Ten-eleven translocase (TET) family of dioxygenases, although the mechanism is far from intuitive: iterative oxidation of 5meC to hydroxymethylcytosine (5hmC), formylcytosine (5fC), and finally carboxylcytosine (5caC) will trigger the thymine DNA glycosylase-dependent base excision repair (TDG BER) pathway to replace modified cytosines with unmodified versions (Kriaucionis and Heintz, 2009; Tahiliani et al., 2009; He et al., 2011; Maiti and Drohat, 2011; Weber et al., 2016). Oxidized forms of 5meC also impede DNA methylation maintenance (Hashimoto et al., 2012; Otani et al., 2013; Ji et al., 2014), thus in that sense, TET proteins contribute to passive DNA demethylation, as well.

The extent to which the TET enzymes contribute to the global demethylation exhibited during embryonic 5meC reprogramming remains an active, and somewhat controversial, area of research. Soon after fertilization, the DNA within the paternal pronucleus becomes strongly enriched for TET3-dependent 5hmC, relative to its maternal counterpart (Gu et al., 2011; Iqbal et al., 2011; Wossidlo et al., 2011). Moreover, paternal DNA methylation is rapidly erased in the zygote, before passive dilution can even occur (Mayer et al., 2000; Oswald et al., 2000). Therefore, it seems quite logical to posit that TET3 is responsible for demethylating the paternal genome. However, careful genetic dissection indicates that this is not entirely the case (Amouroux et al., 2016). Instead, TET3 activity may help protect the paternal genome from DNMT3A-dependent *de novo* DNA methylation (Amouroux et al., 2016; Albert et al., 2020). Of course, this would then imply there is an undiscovered mechanism of active DNA demethylation that must be occurring in the zygote. It should also be noted that in the early embryo, TET3 activity can lead to a passive loss of DNA methylation, independently of base-excision repair (Guo et al., 2014; Shen et al., 2014).

**Figure 1:**
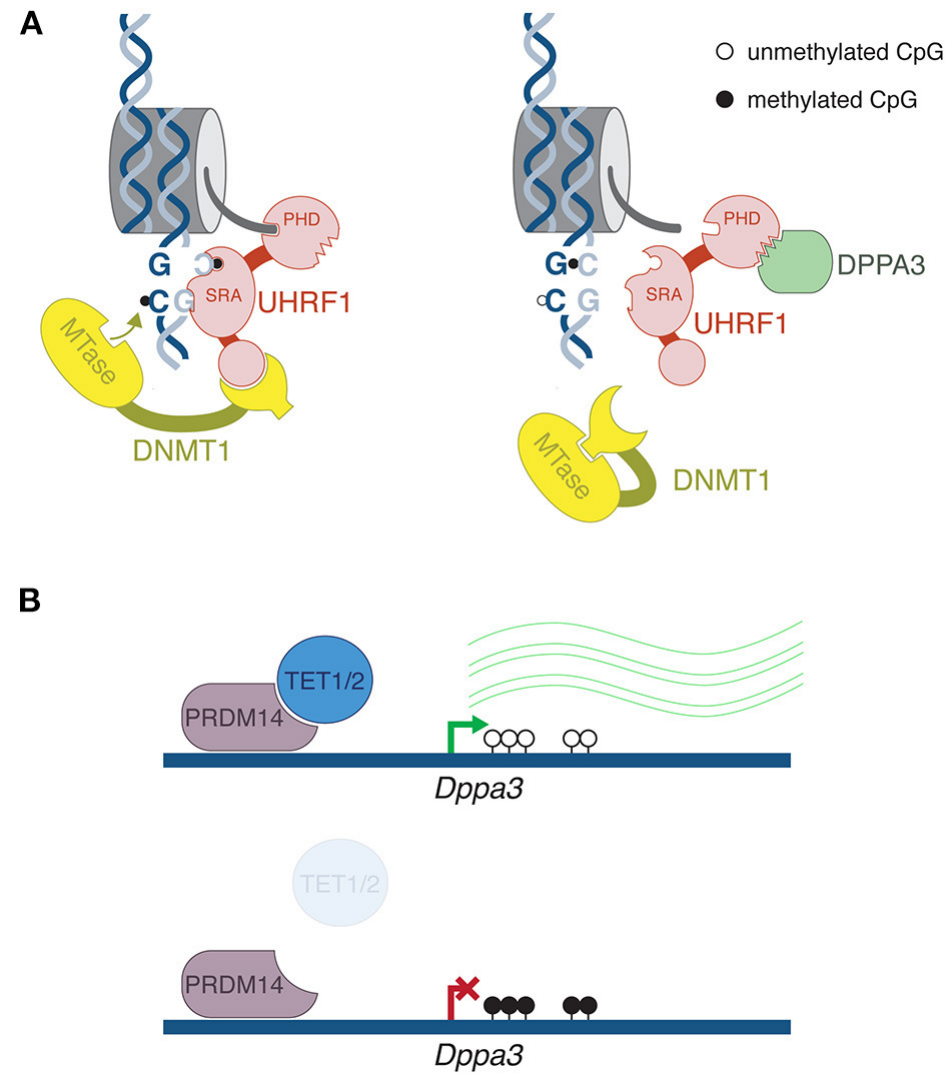
Mechanism of DPPA3-mediated DNA demethylation. **(A)** After DNA replication, the SRA domain of UHRF1 binds to hemimethylated CpG, flipping out the methylated cytosine. Recruitment of DNMT1 directs methylation of the symmetrical unmethylated cytosine on the daughter strand. Crucially, UHRF1 must bind to histone H3 through its PHD domain. DPPA3 binds to the PHD domain, disrupting UHRF1's interaction with chromatin leading to impaired DNA maintenance methylation. **(B)** Top panel: In mouse ESCs, PRDM14 recruits TET1 and TET2 roughly 2 kb upstream of the *Dppa3* promoter, leading to its demethylation and activation. Bottom panel: in *Tet1/2* double mutants, increased DNA methylation at the *Dppa3* gene corresponds with repression. Thus, active demethylation of this one gene is required for global passive demethylation. This mechanism likely does not occur during embryonic DNA methylation reprogramming, but may help explain the wave of DNA demethylation that occurs in the male and female germlines.

In fact, impaired DNA methylation maintenance plays an undeniably important role in reprogramming during the first cell divisions of preimplantation development. The maintenance pathway primarily consists of two proteins: the methyltransferase DNMT1 and its co-factor Ubiquitin-like, containing PHD and RING finger domains, 1 (UHRF1, also known as NP95). While there are many intricate layers to UHRF1/DNMT1 regulation, at its most fundamental level the mechanism is elegantly simple: UHRF1 recognizes hemimethylated CpG sites after DNA replication, and recruits DNMT1 to methylated the cytosine on the daughter strand (Bostick et al., 2007; Sharif et al., 2007) (Figure 1).

In the past few years, studies have emerged that show UHRF1 disruption may be an important means of DNA demethylation. On the spectrum of differentiated cell types, oocytes are quite DNA hypomethylated: roughly 50% of CpGs are methylated in mouse and human oocytes, compared to 70–80% in somatic tissues (Kobayashi et al., 2012; Shirane et al., 2013; Okae et al., 2014). Recently it was demonstrated that the protein Developmental Pluripotency-Associated 3 (DPPA3, also known as STELLA and PGC7) shuttles UHRF1 to the cytoplasm of mouse oocytes (Li et al., 2018a). In the *Dppa3* KO oocytes, UHRF1 returns to the nucleus, and oocyte methylation is increased (Li et al., 2018a; Han et al., 2019). It has been known for decades in mouse preimplantation embryos, an oocyte-specific isoform form of DNMT1 (oDNMT1) is enriched in the cytoplasm, hence its access to DNA is restricted (Carlson et al., 1992; Howell et al., 2001). While immunofluorescence indicates that oDNMT1 still remains largely cytoplasmic in *Dppa3* mutants, a proportion does enter the nucleus as well—that is, DPPA3 activity is the best explanation for the lowly methylated state of the oocyte genome.

Our understanding of the DPPA3 regulation of UHRF1 has been greatly elucidated in a mESC system. When cultured in serum-free conditions and in the presence of MAPK and GSK3β inhibitors (2i), mESCs exhibit globally low levels of DNA methylation—more or less on par with the ICM cells from which ESCs are derived (Ficz et al., 2013; Habibi et al., 2013; Leitch et al., 2013). It was demonstrated that the underlying reason for such low methylation was impaired UHRF1 activity (von Meyenn et al., 2016). While initially UHRF1 was shown to be unstable at the post-translational level in 2i ESCs (von Meyenn et al., 2016), subsequently it was demonstrated that DPPA3 is the factor that is impeding UHRF1 function (Mulholland et al., 2020). Through a rigorous set of biochemical and microscopy-based experiments, Mulholland et al. showed that DPPA3 binds to UHRF1, thus impairing the latter's ability to bind to chromatin (Figure 1). In the mESC system, *Dppa3* mutants are marked by reduction of UHRF1 localization to the cytoplasm, reminiscent of the phenotype in oocytes. However, this aspect of DPPA3-mediated UHRF1 regulation is much less drastic in the cell culture system, thus it is not completely clear if the mechanism of action is exactly the same between oocytes and mESCs. Moreover, DPPA3 can lead to demethylation without its nuclear export function (Du et al., 2019). Regardless, like in oocytes, absence of DPPA3 leads to an increased nuclear fraction of UHRF1 and a gain of DNA methylation. Consistently, when DPPA3 is overexpressed in other cell culture systems, there is a decrease in DNA methylation (Funaki et al., 2014). It should be noted that in addition to DPPA3 action, a number of features of 2i-cultured mESCs likely contribute to the globally DNA hypomethylated state, such as TET protein activity, *de novo* DNA methyltransferase repression (Leitch et al., 2013), and a chromatin state refractory to DNA methylation (van Mierlo et al., 2019), to name three pertinent examples. However, DPPA3 has clearly emerged as a dominant player for this particular feature.

Does the role of DPPA3 in mESCs reflect a role in the DNA demethylation occurring in preimplantation development? While formal demonstration awaits, there are some intriguing indications that indeed DPPA3 performs an analogous function *in vivo*: during normal development, expression of certain classes of TEs are important for activation of the proper embryonic transcription program (Macfarlan et al., 2012; Ishiuchi et al., 2015; Jachowicz et al., 2017); *Dppa3* mutant mice exhibit repression of at least a proportion of these elements at the 2-cell stage (Huang et al., 2017). Given the important role of 5meC in transposon silencing, it is not outlandish to suggest that maintenance of DNA methylation on TEs at the heart of the *Dppa3* transposon expression phenotype, with the caveat that it is difficult to tease apart the maternal from the embryonic effect, given these embryos were generated from *Dppa3* mutant oocytes. Moreover, it should be noted that DPPA3 has been reported to *protect* the maternal genome from TET3-mediated demethylation in zygotes, i.e., the inverse phenomenon (Nakamura et al., 2007, 2012; Han et al., 2019). While not trivial given the severe phenotype of *Dppa3* maternal/zygotic mutants (Payer et al., 2003), hopefully future work will help resolve these apparently contradictory functions.

Finally, Mulholland et al. showed that TET1 and TET2 are required for demethylation of *Dppa3* regulatory regions, thus its activation (Figure 1) (Mulholland et al., 2020). In other words, targeted demethylation of one gene supports global passive demethylation. While this is a compelling finding, it is likely not the mechanism occurring after fertilization. Firstly, DPPA3 is already present in the maternal store of protein inherited from the oocyte (Li et al., 2018a); secondly, TET3 is the active TET enzyme after fertilization, not TET1 or 2; and thirdly, the *Dppa3* gene arrives unmethylated from the oocyte, thus does not require further demethylation (Wang et al., 2014). However, this indeed might be the mechanism to activate *Dppa3* prior to the germline DNA demethylation program. *Dppa3* is expressed in the early stages of germline specification, and importantly, *in vivo* genetic analyses have revealed a role for DPPA3-mediated demethylation in PGCs, although the link with UHRF1 was not made (Nakashima et al., 2013). Incidentally, it has been reported that UHRF1 is downregulated during PGC progression at both the RNA and protein level (Kagiwada et al., 2013; Ohno et al., 2013). It will be necessary to eventually demonstrate if the DPPA3 phenomenology observed in mESCs occur at the relevant stages of *in vivo* development.

## Always Wear Protection: Keeping Developmental Genes DNA Methylation Free

Shortly after reaching its lowest global levels of DNA methylation, the embryo implants into the uterine wall, which coincides with upregulation of the *de novo* DNA methylation program. In a few short days, the genome becomes highly DNA methylated. DNMT3A and DNMT3B show preference for histone 3 lysine 36 di- and trimethylation (H3K36me2/3)-marked regions, respectively, via binding by their PWWP domains (Dhayalan et al., 2010). H3K36me2/3 are broadly deposited in the genome, and may serve to enhance DNMT3A/B activity (Baubec et al., 2015; Weinberg et al., 2019). In general though, the *de novo* methyltransferases exhibit very little discrimination for genomic compartments, with one key exception: CpG island (CGI) promoters, which are distinguished by their markedly high CpG content. Roughly two-thirds of promoters are CGIs, and comprise most housekeeping and developmental genes (Gardiner-Garden and Frommer, 1987; Larsen et al., 1992; Ku et al., 2008). There is nothing about the sequence, *per se*, that should repel *de novo* DNA methylation; in fact, DNMT3A/B show a preference for CpG-rich sequences (Baubec et al., 2015). However, keeping promoters free of methylation is absolutely crucial for proper cellular function; DNA methylation is a very stable epigenetic mark, and ectopic promoter methylation can lead to long-term silencing of important genes. The ADD domain, also harbored by both enzymes, is repelled by H3K4 methylation (Ooi et al., 2007; Otani et al., 2009; Zhang et al., 2010; Guo et al., 2015). Given that H3K4me3 is strongly linked with active promoters, therein lies a simple mechanism to protect promoter sequences from DNA methylation deposition.

During the dramatic flux of DNA methylation in embryogenesis, there must be a means by which genes that are not expressed during reprogramming do not become unwilling targets of DNMT3A/B. Several years ago the discovery was made in mESCs that a large proportion of developmental gene promoters are “bivalently” marked—that is marked by both H3K4me3 and the polycomb repressive complex 2 (PRC2)-deposited H3K27me3 (Azuara et al., 2006; Bernstein et al., 2006). Also known as poised genes, these genes are silent, thanks to PRC2-mediated repression, but at the same time protected from DNA methylation and ready to activate upon the proper developmental cue. Moreover, there is some evidence that H3K27me3 marked regions may be refractory to *de novo* methylation independently of the H3K4 mark (Greenberg et al., 2017; Li et al., 2018b). Finally, TET1 is enriched at bivalent gene promoters (Manzo et al., 2017; Gu et al., 2018). Incidentally, TET1 contains a CxxC domain, which binds specifically to unmethylated CpG-rich sequences, however this domain does not appear to determine TET1 CGI localization (Zhang et al., 2016). Both TET proteins and KDM2B—a CxxC-domain containing complex associated with PRC1—have been demonstrated to protect CGIs from *de novo* DNA methylation (Boulard et al., 2015; Verma et al., 2018). Thus, there are several layers of regulation to prevent DNA methylation-based silencing.

**Figure 2:**
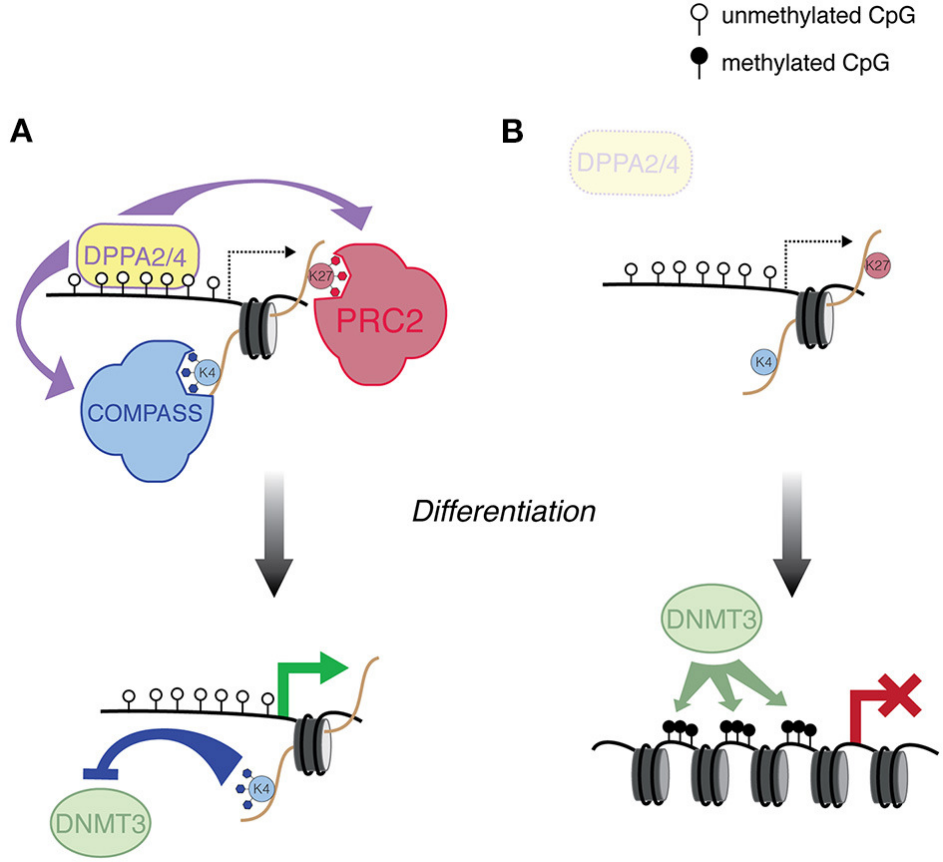
DPPA2/4-mediated regulation of developmental genes. **(A)** DPPA2/4 bind a subset of developmental genes, and recruit the COMPASS and PRC2 complexes, which deposit H3K4me3 and H3K27me3, respectively. These genes are then poised to activate in the proper developmental context. The H3K4me3 protects this class of promoter from *de novo* DNA methylation. **(B)** In *Dppa2/4* mutant ESCs, genes that are normally targeted by DPPA2/4 are susceptible to aberrant *de novo* DNA methyltransferase activity, preventing the ability of this class of genes to activate during differentiation.

In addition to TET and polycomb action, there must be a sequence-based recruitment of H3K4 methyltransferase complexes, i.e., through transcription factors. CGIs serve as important platforms of transcription factor binding, which is associated with alterations in DNA methylation level (Lienert et al., 2011; Krebs et al., 2014). Indeed, integration of a number of genome-wide datasets indicated the main determinant of sequences that do not exhibit DNA methylation is transcription factor binding (Kremsky and Corces, 2020). Recently, two studies converged on two factors that play a role in protecting bivalent genes: DPPA2 and DPPA4 (Eckersley-Maslin et al., 2020; Gretarsson and Hackett, 2020). While both studies utilized an mESC system, the discoveries were through different means. DPPA2/4 are heterodimeric transcription factors that are known to play a role in zygotic genome activation (De Iaco et al., 2019; Eckersley-Maslin et al., 2019; Yan et al., 2019), but they also are bound to bivalent genes (Engelen et al., 2015; Hernandez et al., 2018; Klein et al., 2018). Intriguingly, *Dppa2/4* mutant mice exhibit developmental defects far after the embryonic stages where developmental genes are enriched for bivalent marks (Madan et al., 2009; Nakamura et al., 2011). Following up on this curiosity, Eckersley-Maslin et al. showed that in *Dppa2/4* mutant mESCs, a subset of DPPA2/4 targets lose both H3K4me3 and H3K27me3 enrichment, indicating a role for these transcription factors in recruiting both silencing and activating complexes (Eckersley-Maslin et al., 2020) (Figure 2). Moreover, during differentiation to embryoid bodies, this subset of genes acquire DNA methylation and fail to activate, likely because they no longer are protected from *de novo* DNA methyltransferases (Figure 2).

Gretarsson and Hackett ultimately arrived at similar conclusions, although they discovered a role for DPPA2/4 through different means. Using a clever mESC-based CRISPR screen, they searched for mutants that failed to activate a methylated reporter during an *in vitro* model of global DNA demethylation (Gretarsson and Hackett, 2020). *Dppa2/4* were two of the top hits from the screen, but mutations did not lead to global DNA methylation perturbations. Consistent with the study from Eckersley-Maslin, DNA methylation abnormalities were restricted to a proportion of bivalently marked gene promoters. Curiously, DPPA2/4 also target LINE1 retrotransposons, although their role in transposon regulation is less clear. It should be noted that DNA methylation defects do not occur at all DPPA2/4 targets in the double mutant background; moreover, there are bivalent promoters that are not DPPA2/4 targets. That is all to say, DPPA2/4 are only two of the transcription factors important for DNA methylation protection during epigenetic reprogramming and there is a high probability others exist. Nevertheless, these are two important studies that undergird a model wherein sequence-specific transcription factors not only prime genes for proper expression during development, but also play a protective role against ectopic silencing.

## Concluding Remarks: DPPA3, Not Just A Basic Protein

DNA methylation, if misregulated, can have dire consequences. As discussed above, the improper *de novo* DNA methylation of promoters can lead to stable silencing of key developmental regulators. Perhaps more worrisome, the absence of DNA methylation can result not only in the ectopic expression of protein coding genes, but massive transposon derepression, which can have far ranging and deleterious effects (Walsh et al., 1998). Mouse embryos lacking either *de novo* or maintenance DNA methylation machinery die shortly after gastrulation (Li et al., 1992; Okano et al., 1999). Therefore, mammals must have evolved compensatory mechanisms to control the genome during not one, but two waves of DNA methylation reprogramming (Walter et al., 2016; Hill et al., 2018). What is more, the lowest levels of DNA methylation occur in pluripotent stem cells and primordial germ cells—the cell types that give rise to all somatic tissues and the germline, respectively. It is difficult to imagine cells that are more important for proper organismal development and transmission of genetic material to the next generation. Why then do mammals take such extraordinary apparent risks with their genome? Even among vertebrates this phenomenon is odd: zebrafish, by comparison, undergo nothing so drastic with their methylome during embryonic development (Skvortsova et al., 2019).

In investigating the evolutionary conservation of the *Dppa3* gene, Mulholland et al. revealed it is not found outside mammals. Amazingly, when the mouse DPPA3 protein was incubated with the egg extracts of the amphibian *Xenopus laevis*, the mouse protein inhibited the frog UHRF1. Furthermore, when the fertilized eggs from the model fish medaka were injected with *Dppa3* mRNA, the embryos exhibited dramatic hypomethylation (Mulholland et al., 2020). Therefore, DPPA3 has evolved as a potent DNA demethylation factor that can disrupt UHRF1 function in distant species. The authors suggest that perhaps *Dppa3* arose in the mammalian lineage in concert with the role of transposon expression regulating the early transcription program. This certainly is possible, however it does not explain why global demethylation occurs exclusively in mammals, and also why it happens twice in development.

It is notable that *Dppa3* is only present in placental mammals; marsupials and egg-laying monotremes lack the gene. Classic experiments in the 1980s demonstrated that mouse paternal and maternal genomes are not equivalent—androgenetic diploids exhibit robust placenta, whereas conversely gynogenetic diploids harbor severely undersized placenta (Surani and Barton, 1983; Barton et al., 1984; McGrath and Solter, 1984; Surani et al., 1984). This result is consistent with genetic conflict theory, which states that there is a conflict between the parental genes with regards to offspring development (Moore and Haig, 1991). In this case, the paternally expressed genes promote larger placenta leading to greater resource allocation to the developing embryo and fetus at the mother's expense, and for the maternally expressed genes it is the inverse. This theory was developed to explain the existence of genomic imprinting, which is parent-specific gene expression controlled by DNA methylation patterns inherited from the gametes. Pertinently, neither androgenetic nor gynogenetic embryos are viable. Given the stark DNA methylation asymmetry between gametes, perhaps *Dppa3* evolved in order to prevent either the paternal or maternal genome exerting too much control with regards to inherent conflict. Imprint control regions, in turn, evolved mechanisms to escape DNA methylation erasure during embryogenesis. In the case of marsupials, there is evidence of germline reprogramming and genomic imprinting (Renfree et al., 2008; Suzuki et al., 2013; Ishihara et al., 2019), however evidence for embryonic reprogramming in both marsupials and monotremes is limited. Hopefully future studies will interrogate dynamic DNA methylation after conception in non-placental mammals, which will not only provide insights into DPPA3 function, but into evolutionary theory.

## Author Contributions

MVCG wrote the manuscript and prepared the figures.

## Conflict of Interest

The author declares that the research was conducted in the absence of any commercial or financial relationships that could be construed as a potential conflict of interest.
